# Wenlin procedure combined with Wung procedure for treatment of severe pectus carinatum

**DOI:** 10.1093/jscr/rjac537

**Published:** 2022-11-26

**Authors:** Wenlin Wang, Weiguang Long, Yang Liu, Bin Cai, Juan Luo

**Affiliations:** Department of Chest Wall Surgery, Guangdong Second Provincial General Hospital, Guangzhou, China; Department of Chest Wall Surgery, Guangdong Second Provincial General Hospital, Guangzhou, China; Department of Chest Wall Surgery, Guangdong Second Provincial General Hospital, Guangzhou, China; Department of Chest Wall Surgery, Guangdong Second Provincial General Hospital, Guangzhou, China; Department of Chest Wall Surgery, Guangdong Second Provincial General Hospital, Guangzhou, China

## Abstract

Pectus carinatum can be treated with a single minimally invasive surgery, but the severe cases will appear secondary depression after treatment, which needs to be corrected additionally. We used the combined Wenlin procedure and Wung procedure to treat a patient with severe pectus carinatum and achieved satisfactory results.

## INTRODUCTION

Pectus carinatum is a common thoracic deformity, and its main feature is protrusion of anterior chest wall [1, 2]. Generally, patients do not have clear symptoms, but most of them are not satisfied with the appearance of the chest wall, and some patients will have psychological problems because of this. Therefore, most patients have the desire of surgery [1–4]. Early operations were mainly open operations. In recent years, minimally invasive surgery has entered clinical practice [1–4]. Wenlin procedure is a minimally invasive surgery designed by us for pectus carinatum [1, 2]. We used it in a large number of patients and achieved satisfactory results [5]. However, in the operation of extremely serious pectus carinatum patients, local depression may occur after Wenlin procedure, which needs further treatment. We use Wung procedure for surgery correction, and generally get good results [6–9].

## CASE REPORT

**Figure 1:**
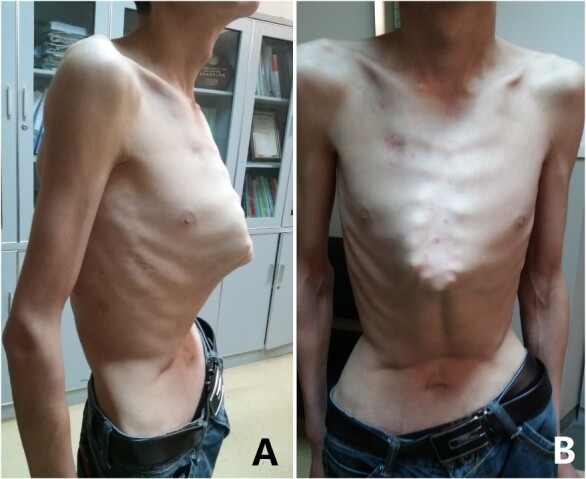
Appearance of chest wall before operation. (**A**) Lateral view; and (**B**) front view.

**Figure 2:**
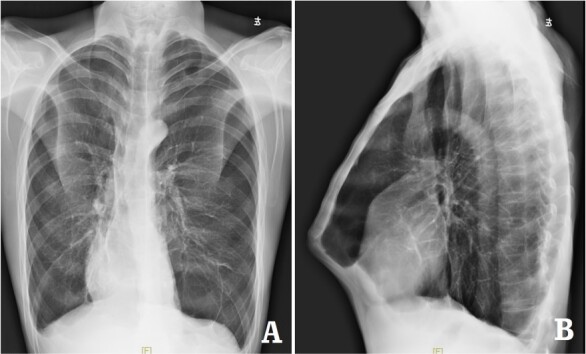
X-ray examination before operation. (**A**) Posteroanterior radiograph; and (**B**) lateral radiograph.

**Figure 3:**
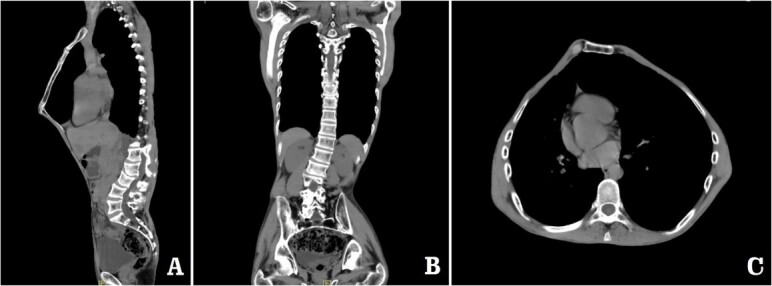
Computed tomography examination before operation. (**A**) Sagittal view; (**B**) coronal view; and (**C**) sectional view.

**Figure 4:**
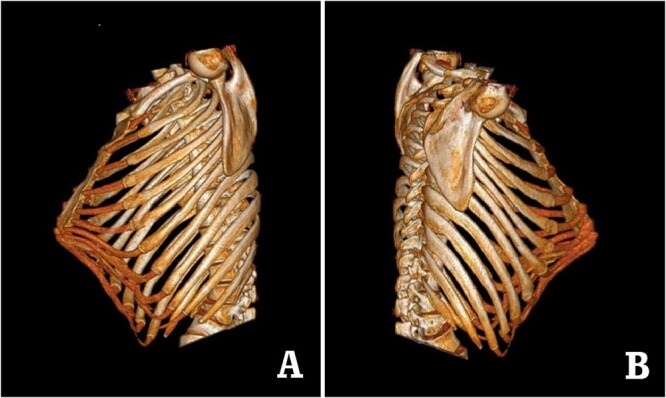
Preoperative 3D reconstruction pictures. (**A**) Left side view；and (**B**) right side view.

**Figure 5:**
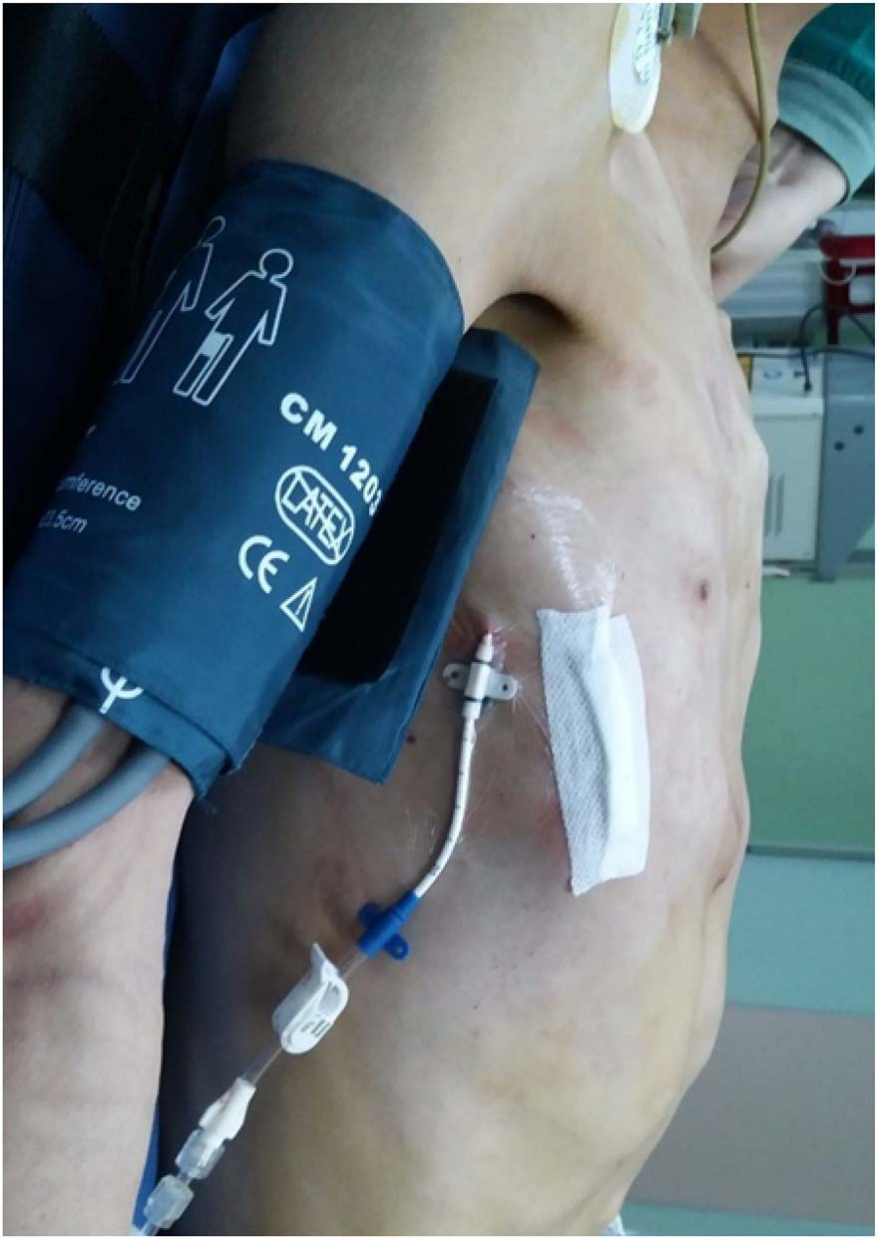
Appearance of chest wall after operation.

**Figure 6:**
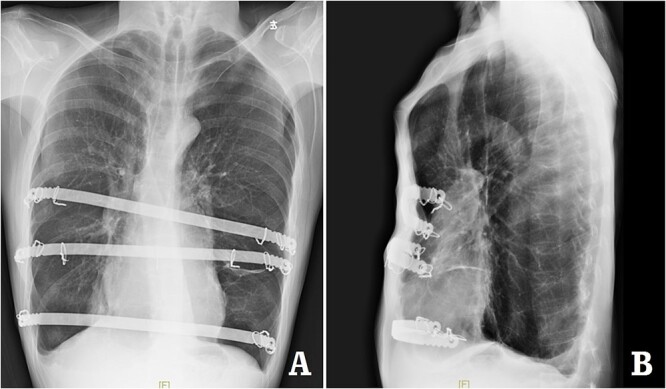
X-ray examination after operation. (**A**) Posteroanterior radiograph; and (**B**) lateral radiograph.

**Figure 7:**
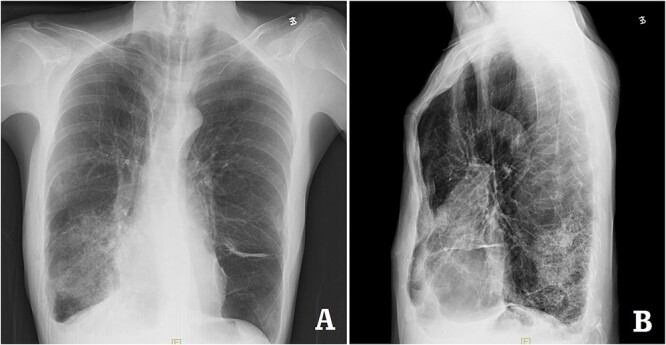
X-ray examination after the bars were taken out. (**A**) Posteroanterior radiograph; and (**B**) lateral radiograph.

The patient was a 29-year-old male. He was found to have protrusive deformity on the anterior chest wall since childhood. The deformity was not serious in early years, but worsened after adolescence, with the lower part of the sternum protruding at an acute angle. At the age of 25, he developed right spontaneous pneumothorax and received surgical treatment at local hospital, but his thoracic deformity was not treated. As the deformity continued to worsen, which seriously affected the appearance of the chest wall, the patient was admitted to our hospital for surgery. Preoperative physical examination showed that the anterior chest wall was protrusive seriously, with a sharp tip protruding forward. The rib arches on both sides were slightly depressed (Fig. 1). Imaging examination showed that the anterior chest wall was protrusive, and the lower end of the sternum was at the forefront of the protrusion. His heart moved to the right, and the rib arches were slightly depressed (Figs 2–4). The operation was performed under general anesthesia. Two longitudinal incisions were made on both sides of the chest wall respectively. The incisions were located between the front axillary line and the median axillary line, with the length of ~5 cm. The chest wall muscles were dissected to expose the ribs in the incisions. Two tunnels were made on the highest plane of the protrusion, with interval of 3 cm. The tunnels were located in the deep layer of the chest wall muscles and bone structures. Two steel bars were inserted into the tunnels to flatten the front protrusion with their median parts, and then, both ends of the steel bars were fixed on the ribs at the lateral chest wall. The above operation was the main content of Wenlin procedure [1, 2, 5]. After this procedure was completed, the lower part of the chest wall showed obvious depression, especially in the middle of the rib arches. Then, Wung procedure was performed [6]. A third tunnel was made at the plane passing the midpoint of the rib arch, which passes through the bilateral thoracic cavity. The third steel bar was inserted into the tunnel. After the steel bar was rotated and fixed to the ribs, the depression was supported totally. The incisions were closed, and the operation was completed. The deformity of anterior chest wall disappeared completely after the operation (Fig. 5). The operation time was 75 min. The intraoperative bleeding volume was 40 ml. Postoperative X-ray examination showed that the bars position was normal (Fig. 6). He was discharged 7 days after operation. Follow up for 1 year showed satisfactory recovery. The steel bars were taken out 1 year after the operation, and the appearance of the thorax was normal and there was no recurrence (Fig. 7).

## DISCUSSION

There are many techniques for pectus carinatum surgery [1–5]. Each technique has different characteristics. Wenlin procedure is a special operation designed by us for pectus carinatum [1, 2, 5]. This operation is a simple and convenient technique. For the less serious deformities of pectus carinatum, this procedure alone can achieve satisfactory results. However, for patients with severe pectus carinatum, after the protrusion is pressed, secondary depression will occur. Such depression will not only affect the effect of surgery, but also affect the function of the heart or lungs, so it is necessary to be corrected [7–9]. In addition, some pectus carinatum deformities are always combined with a certain degree of depression. After receiving Wenlin procedure, the depression will be significantly aggravated, and they also need to be corrected [7–9].

Currently, the most popular method for chest wall depression is Nuss procedure [10]. However, due to many disadvantages of this operation, many authors have made lots of improvement about it. We have also done similar work. The modified surgery we designed is Wung procedure [6]. Although this procedure still uses steel bar to support the depression, the specific operation details are completely different. One of the most obvious differences is that we use a special steel bar fixation method, namely Wang technique [11]. The difference of surgical details makes this procedure have more advantages. After Wenlin procedure was completed, the depression of this patient was significantly deepened. We directly support the depression with Wung procedure, and the depression was easily corrected.

This patient is the most serious case among all our patients with pectus carinatum. If only one method is used for correction, it is difficult to obtain good results. Because we have used two kinds of suitable operations at the same time, the operation is not only simple and easy, but also obtains a very perfect effect.

Our clinical experience shows that secondary depression often occurs after the protrusion deformity is flattened. If such depression is not treated, the surgical effect will be affected. The combination of Wenlin procedure and Wung procedure can completely eliminate the protrusion and secondary depression, so it is an ideal choice for the treatment of severe pectus carinatum [7–9].

## CONFLICT OF INTEREST STATEMENT

None declared.

## FUNDING

None.
